# Brain organoid model systems of neurodegenerative diseases: recent progress and future prospects

**DOI:** 10.3389/fnins.2025.1604435

**Published:** 2025-05-23

**Authors:** Saniyah Shaikh, Luqman Siddique, Hafsah T. Khalifey, Rutaba Mahereen, Thaabit Raziq, Rushdan M. Firdous, Aisha Siddique, Ismail M. Shakir, Zara Ahmed, Arshiya Akbar, Eman A. Alshehri, Raja Chinappan, Alaa Alzhrani, Tanveer Ahmed Mir, Ahmed Yaqinuddin

**Affiliations:** ^1^College of Medicine, Alfaisal University, Riyadh, Saudi Arabia; ^2^Bioscience Program, Biological and Environmental Science and Engineering Division (BESE), King Abdullah University of Science and Technology (KAUST), Thuwal, Saudi Arabia; ^3^KAUST Center of Excellence for Smart Health (KCSH), Thuwal, Saudi Arabia; ^4^Shadan Degree and P.G. College for Women, Osmania University, Hyderabad, India; ^5^Tissue/Organ Bioengineering and BioMEMS Lab, Organ Transplant Centre of Excellence (TR&I Dept), King Faisal Specialist Hospital and Research Centre, Riyadh, Saudi Arabia; ^6^Department of Medical Laboratory Technology, Faculty of Applied Medical Sciences, King Abdulaziz University, Jeddah, Saudi Arabia

**Keywords:** brain, organoids, stem cells, neurodegenerative disorders, disease modeling, Parkinson’s disease, Alzheimer’s disease, Huntington’s disease

## Abstract

Neurological diseases are a leading cause of disability, morbidity, and mortality, affecting 43% of the world’s population. The detailed study of neurological diseases, testing of drugs, and repair of site-specific defects require physiologically relevant models that recapitulate key events and dynamic neurodevelopmental processes in a highly organized fashion. As an evolving technology, self-organizing and self-assembling brain organoids offer the advantage of modeling different stages of brain development in a 3D microenvironment. Herein, we review the utility, advantages, and limitations of the latest breakthroughs in brain organoid endeavors in the context of modeling three of the most prevalent neurodegenerative diseases—Alzheimer’s, Parkinson’s, and Huntington’s disease. We conclude the review with a perspective on the future prospects of brain organoid models with their myriad possible applications in translational medicine.

## 1 Introduction

The brain is one of the most essential organs that control and regulate all the critical bodily functions including maintaining the senses, voluntary or involuntary movements, behavior, emotions, and memories. Elucidating its basic growth stages and developmental mechanisms has been a major challenge for the research community over the past several decades. The increasing number of individuals with neurodegenerative as well as cognitive disorders caused by various diseases such as Alzheimer’s disease (AD), Parkinson’s disease (PD), and Huntington’s disease (HD) has become a major healthcare concern (Mir and Shinohara, 2013; Trojsi et al., 2018; Akula et al., 2023; Ogonah et al., 2025). Therefore, intensive research attempts are being made to dissect and investigate the developmental mechanisms of neuronal diseases or neurogenesis using advanced *in vitro* disease detection and modeling technologies (Mir et al., 2011; Di Lullo and Kriegstein, 2017; An et al., 2024). Toward that end, human brain organoids technology has revolutionized the field of neuroscience with their ability to mimic several aspects of the developing brain, *in vitro*. The remarkable three-dimensional structure of the organoid, which resembles the complexity of the human brain, offers great promise for better understanding and modeling the development of neurological diseases. Attempting to recapitulate the diversity of cell-types and cell-building organization of the embryonic human brain, researchers have successfully generated neural tissue—termed human brain organoids—using human pluripotent stem cells (hPSCs), including embryonic stem cells (ESCs) and induced pluripotent stem cells (iPSCs) (Bhaduri et al., 2020; Jacob et al., 2020; Fedorchak et al., 2021). These self-organized 3D structures continue to provide new insights into basic biology by mirroring the developmental pathways of the human brain with great accuracy (Lee et al., 2017; Zhang et al., 2022). Overall, the organoid models provide a unique opportunity to explore the physiological niches and environmental complexities of the developing human brain and to unravel the enigmatic causes behind associated abnormalities. By precisely replicating key brain structures like the midbrain, hippocampus, pituitary, hypothalamus, and cerebellum, these organoids provide an invaluable model for better understanding the mechanisms of neurodevelopmental conditions and may facilitate detailed investigation of many other unexplored pathogenetic mechanisms leading to neurodegeneration and dementia-linked disorders (Shou et al., 2020).

This review provides an overview of common neurological disorders and highlights recent progress and advancements in the field of brain organoids. In particular, it sheds light on the employment of various cell sources and emerging technologies for establishing brain organoids to more effectively reproduce the three-dimensional microenvironment of the brain. In conclusion, the current limitations and future potential of these models and their eventual translational applications are discussed.

## 2 Methodology

A comprehensive search was conducted, screening through multiple databases, which included PubMed, Web of Science, and SCOPUS, and identified relevant articles published between the years 2018 and 2024. The search strategy combined Medical Subject Headings (MeSH) with text keywords using Boolean operators (OR and AND). The final search strategy developed was as follows:

(“Heredodegenerative Disorders, Nervous System”[Mesh] OR “Lewy Body Disease”[Mesh] OR “Striatonigral Degeneration”[Mesh] OR “Nerve Degeneration”[Mesh] OR “Basal Ganglia Diseases”[Mesh] OR “Frontotemporal Dementia With Motor Neuron Disease” [Supplementary Concept] OR “Corticobasal Degeneration”[Mesh] OR “Neurodegenerative Diseases”[Mesh]) OR (“Alzheimer’s*” [tw] OR AD [tw] OR “Parkinson’s*” [tw] OR Parkinsons OR “Huntington’s*” [tw] OR Huntingtons* OR “Amyotrophic lateral sclerosis” [tw] OR ALS [tw] OR “Frontotemporal dementia” [tw] OR FTD [tw] OR “Spinocerebellar degeneration” [tw] OR SCD [tw] OR “Leigh syndrome” [tw] OR “Multiple sclerosis” [tw] OR MS [tw] OR Tauopathies [tw] OR CJD [tw] OR “Creutzfeldt-jakob disease” [tw] OR Protein aggregation [tw] OR Beta amyloids [tw] OR Striatal pathways [tw] OR lewy body* [tw] OR Neuronal loss [tw] OR Impaired synaptic transmission [tw] OR Neuroinflammation [tw]) AND (Brain Organoid*) OR (3D bioprinted organoid) OR (Microfluidic organ-on-a-chip system) OR (Co-culture organoid) OR (Midbrain organoid) OR (Cerebellar organoid) OR (Spinal cord organoid) OR (Brain assembloid) AND (pathophysiology OR disease Modeling OR disease mechanisms OR pathology OR therapeutic strategies OR drug screening OR gene therapy OR limitations OR challenges).

The inclusion and exclusion criteria for this review were based on disease, intervention, and outcome parameters. Studies focusing on neurodegenerative diseases using (NDs) brain organoid models, specifically those addressing disease modeling, pathological hallmarks, functional deficits, and therapeutic interventions, were considered. Experimental studies of various types were eligible for inclusion, including randomized controlled trials, cohort studies, case-control studies, case reports, observational studies, reviews, and those with and without a control group.

Conversely, studies were excluded if they only involved cell cultures without brain organoids, focused solely on brain organoid models in animal systems, or investigated interventions unrelated to the modeling or understanding of NDs in brain organoids. Moreover, studies that lacked relevant outcome measures pertinent to NDs or that focused solely on outcomes related to non-neurological conditions were excluded.

## 3 Overview of common neurological disorders

Neurodegenerative diseases (NDs) are characterized by decreased functional activity of neurons and degeneration of neuronal cells or their terminal branches, resulting in deterioration and eventually death. These NDs are generally classified based on their characteristic symptoms, depending on the brain regions affected during the progression or gradual loss of neurons and synaptic connections. Common NDs include progressive neuropsychiatric disorders such as AD, PD, and HD, among others.

### 3.1 Alzheimer’s disease

AD is one of the most common multifaceted NDs, affecting millions of people worldwide in basic mental functions. Neuropathologically, it is characterized by extracellular deposition of amyloid-beta (Aβ) plaques and intracellular neurofibrillary tangles composed of abnormally hyperphosphorylated tau protein. These abnormalities disrupt neuronal communication, leading to synaptic dysfunction, neuroinflammation, and widespread neuronal loss, which, among other symptoms, ultimately culminate in cognitive decline and memory impairment (Knopman et al., 2021). AD has been documented to present as either sporadic AD (SAD) or familial AD (FAD). SAD is more common and is influenced by the complex interactions between genetic and environmental factors, and FAD is caused by genetic mutations in the amyloid precursor protein (APP) and presenilin (PSEN1 and PSEN2) genes (Bi et al., 2021). Aging is the most significant risk factor contributing to the development of SAD. With time accelerating the disease’s pathological processes, there is impaired clearance of Aβ, oxidative stress, mitochondrial dysfunction, and a weakened blood–brain barrier (BBB) (Faravelli et al., 2020; Bi et al., 2021). Key genetic risk factors, such as the APOE4 allele, also play a critical role in worsening Aβ buildup and tau pathology, highlighting the complex molecular mechanisms driving the disease (Knopman et al., 2021).

### 3.2 Parkinson’s disease

PD is the second most common ND, affecting 2 in 1,000 people, typically over the age of 65 (McComish et al., 2022; Souza et al., 2023). PD is pathologically characterized by tremor, rigidity, bradycardia, and unstable postural appearance in some patients as the disease progresses due to a decrease in dopaminergic neurons in the substantia nigra of the midbrain. Impairment in the autophagic system of the neurons leads to α-synuclein accumulation and formation of cytoplasmic inclusions. Such aggregates cause neuronal symptoms like loss of spontaneous motor activity, postural instability, resting tremors, and muscular rigidity with bradykinesia (Chang et al., 2020; Souza et al., 2023). Genetic predisposition of LRRK2 (leucine-rich repeat kinase two gene locus), PARK1 gene (encodes α-synuclein protein), PARK2 (encodes parkin), PARK6 for PINK1 (PTEN-induced kinase 1), PARK7, and PARK8 (encodes LRRK2 protein) mutations increase its susceptibility. To better understand the dynamics of these mutations associated with PD, 2D cultures and models were initially used. However, their ability to accurately replicate cellular functions proved to be limited. In response, 3D models and organoids emerged as more promising alternatives, offering improved results (Souza et al., 2023).

### 3.3 Huntington’s disease

HD is an autosomal-dominant inherited ND caused by an increased number of long cytosine-adenine-guanine (CAG) trinucleotide repeats in the Huntingtin (HTT) gene, eventually leading to dysfunction of the striatum and cortex. Despite ongoing advancements in research and therapy, no definite treatment currently exists to alter the disease’s progression. In the pursuit of understanding this ND, Conforti et al. (2018) utilized organoids derived from HD-iPSC lines to model the disease’s impact on neuronal development and function. This approach provided a powerful tool for exploring the pathophysiological mechanisms underlying HD since it focused on several key aspects of neuronal differentiation and organization (Conforti et al., 2018). The pathological findings of HD mainly comprise immature ventricular/subventricular zone differentiation, altered cytoarchitecture and cortical layer organization, as well as impaired genetic pathways related to neuronal migration and differentiation, including the downregulation of a key neurogenic factor, NeuroD1. It has been well-established that the complete loss of wild-type HTT protein results in embryonic lethality, emphasizing its role in early neurodevelopmental stages. Furthermore, both 2D and 3D neuronal models generated from iPSCs with the mutant HTT (muHTT) gene have consistently revealed neurodevelopmental abnormalities directly linked to the expanded repeat mutation (Wray, 2020). Nevertheless, it remains uncertain how early neurodevelopmental defects might influence neuronal function in later stages of life and whether early therapeutic strategies might offer a significant modification in disease progression (Conforti et al., 2018).

## 4 The evolution and innovation in brain organoid technology: from cell source to advanced methods

Essentially there are two distinct approaches for brain organoid generation—unguided or guided differentiation. In the unguided approach, the hPSCs are aggregated into embryoid bodies (EBs) and are then allowed to differentiate with minimal external factors or “guides” into a more heterogeneous structure called cerebral organoids. These cerebral organoids comprise different neuronal and non-neuronal cell types in proportions that do not truly mimic the natural proportions in a human brain. Also, given the differentiation process is unguided, there exists significant batch-to-batch and cell-line-to-cell-line variations limiting reproducibility and derived conclusions (Qian et al., 2019).

The guided approach, on the other hand, involves the use of growth factors and small molecules to control and direct differentiation of the EB, into specific cell-types making the resultant organoid compartment-specific, such as spinal, cortical, cerebellar, midbrain, thalamus, hippocampal, and retinal organoids (Qian et al., 2019; Zhou et al., 2024). Guided brain organoids circumvent the inconsistencies of the unguided approach and allow cell-type specific analyses.

### 4.1 Cell source selection

Organoids can be created from different cell types, such as iPSCs, ESCs, adult stem cells (ASCs), and even tumor cells, each cell source providing different advantages and potential applications as outlined below.

#### 4.1.1 Pluripotent stem cells: iPSCs and ESCs

Directed differentiation of pluripotent stem cells (PSCs) yields PSC-derived organoids. This process involves the formation of distinct germ layers (endoderm, mesoderm, or ectoderm), followed by exposure to particular growth factors, signaling molecules, and cytokines to guide cell-specific differentiation and maturation. These organoids contain a variety of cell types, such as mesenchymal, epithelial, and endothelial cells, which enhances their utility in modeling complex tissues (Brassard and Lutolf, 2019). However, the limited interaction between the different cell types restricts their ability to fully replicate *in vivo* interactions (McCauley and Wells, 2017). iPSCs are generated by the reprogramming of somatic cells, whereas ESCs are obtained from the inner cell mass of the blastocyst. Both iPSCs and ESCs can differentiate into a wide range of cell types, enabling the creation of complex organoid models. iPSC-derived organoids typically resemble fetal tissues in that they hardly reach the adult tissue stage as their ability to proliferate ceases after a certain period (Camp et al., 2015). This makes them excellent models for studying developmental biology and organogenesis (Clevers, 2016). Comparatively, ESC-derived organoids are more mature and can be utilized to study later stages of organ development. However, the ethical concerns surrounding the use of ESCs restrict their application (Liang et al., 2022).

#### 4.1.2 Adult stem cells

ASCs represent specific adult tissue types and serve as an effective model for adult tissue functions. They closely mimic the original tissue, preserving homeostasis and regenerative potential, which makes them highly valuable for regenerative medicine and for modeling diseases like cancer or ND (Kaushik et al., 2018).

#### 4.1.3 Tumor-derived organoids

Tumor-derived organoids (Tumoroids) are organoids derived from tumor tissues, maintaining the genetic and histological features of the original tumor. These are particularly useful for cancer research, especially in studying tumor biology, preclinical testing, and patient-specific treatment (Krieger et al., 2020).

#### 4.1.4 Multilineage organoids and assembloids

Multilineage organoids and assembloids are valuable for simulating the intricate, complex interactions between the different cell types. By combining cells from multiple cell lineages or tissues, these models closely replicate both *in vivo* pathophysiology and physiology. Assembloids are created by co-culturing organoids from different tissues or regions, allowing them to interact as they would in the body.

In the context of brain assembloids, also referred to as fused organoids, recent studies have employed the technique where guided compartment-specific organoids are first generated separately, then brought together in a co-culture along with other specialized cell types (immune cells, endothelial cells) where they fuse forming an assembloid. This method has allowed meticulous generation of complex multi-compartment organoids. This model facilitates exploration and investigation of physiological and pathophysiological cell–cell communication, inter-compartmental signaling, and brain circuitry (Paşca, 2019; Makrygianni and Chrousos, 2021; Pașca et al., 2022). For instance, Bagley et al. (2017) combined cerebral organoids from different regions of the brain to study interactions between brain areas, neuron migration, and long-distance projections. Sloan et al. (2018) fused ventral and dorsal forebrain organoids to study the synaptic integration between the GABAergic and glutamatergic neurons. They extended the application to create assembloids derived from patients with rare forms of autism and epilepsy. Song et al. (2019) termed their assembloids tri-cultured hybrid spheroids where they integrated cortical progenitor cell spheroids with vascular spheroids and mesenchymal stem cells to investigate interactions among various cell types.

### 4.2 Advanced technologies for organoid development

#### 4.2.1 Microfluidics-based organoid culture

Microfluidic chips are compact devices that manage the flow of small amounts of liquid, such as cell culture media, through narrow channels. These chips enable researchers to more precisely control environmental factors around organoids, including fluid flow, nutrient supply, and waste removal, compared to traditional culture methods. By providing dynamic conditions that closely resemble the body’s natural microenvironment, microfluidics support better organoid maturation and functionality (Yin et al., 2016). Microfluidic systems are commonly employed in organ-on-a-chip models, where multiple organoids (like those from the brain, liver, or heart) are cultured together in a single device. This allows for the study of inter-organ interactions and testing of how drugs affect multiple organs simultaneously. The precise environmental control offered by microfluidics makes it especially useful for investigating complex processes such as organ development, disease modeling, and drug testing with improved accuracy. It also allows for the creation of highly reproducible and scalable organoid models, ideal for high-throughput screening (Yang et al., 2023).

### 4.3 Organoid vascularization and perfusion

The vascularization of organoids is a critical development in organoid engineering, addressing the limitations of diffusion-based nutrient and oxygen delivery that hinder their physiological relevance and functional longevity. Advanced methodologies encompass co-culture systems with endothelial cells and supporting stromal cells, such as pericytes, to facilitate *de novo* vasculogenesis and vascular organoid integration to enable pre-formed vascular structures to fuse with lineage-specific organoids (Salmon et al., 2022). Organoid-on-a-chip platforms, utilizing microfluidic technologies, simulate hemodynamic conditions, promoting perfusable vascular networks through controlled fluid shear stresses and mechanical stimuli (Shin et al., 2021; Nwokoye and Abilez, 2024). Additionally, 3D bioprinting with vascular channels and bioinks enriched with growth factors, such as VEGF and FGF-2, permits precise spatial patterning of vasculature within organoid matrices (Nwokoye and Abilez, 2024). These approaches synergize to create perfusable vascular networks that are critical for maintaining cellular homeostasis and reducing hypoxia-induced necrosis in organoids (Nwokoye and Abilez, 2024).

Applications in drug screening are significantly enhanced by vascularized organoids, as they replicate organ-level pharmacokinetics and pharmacodynamics, including drug absorption, metabolism, and clearance processes mediated by vascular barriers, such as the BBB (Nwokoye and Abilez, 2024). The inclusion of vascular structures enables the study of angiocrine signaling, vascular permeability, and endothelial responses under various pharmacological interventions. By incorporating patient-derived cells, vascularized organoids advance personalized medicine by enabling precise drug screening and the prediction of adverse drug reactions (Osaki et al., 2018).

#### 4.3.1 Bioprinting of technology for brain research

Bioprinting is a computerized process that uses 3D printing technology for fabricating living cell-laden constructs in a layered format. Bioprinting encompasses a variety of techniques for creating 3D biological structures. Laser-assisted, inkjet, and extrusion-based methods are the three primary approaches (D’Antoni et al., 2023). Using bioinks (a mixture of cells and materials) allows precise placement of cells in 3D space, enabling the creation of more complex and organized organoids that mimic real organs, making organoids more physiologically relevant (Layrolle et al., 2022). It can also print scaffolds, i.e., supporting cells to grow in the right shape and orientation. Bioprinting enables the creation of complex tissues, like vascular networks (blood vessels), within organoids, which the traditional methods struggle to achieve. It holds great potential, which is crucial for the generation of larger, functional organoids used in drug screening, disease modeling, and transplant research (Ren et al., 2021). Within extrusion-based bioprinting, several specific approaches are commonly used, such as layer-by-layer assembly and sacrificial bioinks (Kolesky et al., 2016; Layrolle et al., 2022). According to D’Antoni et al. (2023), two primary approaches are used for bioprinting stem cells. In the first approach, ESCs, hPSCs, or other stem cells are directly printed into a construct, where their inherent potency drives differentiation into desired cell types. In contrast, the second approach involves pre-differentiating stem cells into specific lineages before incorporating them into the bioprinting process. Each approach offers unique advantages and is selected based on the complexity and purpose of the bioprinted tissue. 3D bioprinting addresses key weaknesses of traditional organoids by allowing for better blood supply to all regions in the living structure, and the methods used in bioprinting allow for more precise control over the spatial arrangement of cells and hence for higher reproducibility in high-throughput drug screening applications (Layrolle et al., 2022). Key components in the fabrication of bioprinted models are scaffolds. Scaffolds are structural frameworks typically made of hydrogels that support the attachment, proliferation, and differentiation of neural cells, getting organoids a few steps closer to mimicking *in-vivo* conditions. One important property conferred by scaffolds is the printed model’s elastic modulus, “a measure of an organ or a hydrogel’s resistance to elastic deformation” (Whitehouse et al., 2023). As per the findings of Bejoy et al. (2018), low elastic moduli (approx. 300 Pa) favor the differentiation of human iPSCs into forebrain-like neurons, and higher elastic moduli (approx. 1 kPa) into hindbrain-like neurons. Ma et al. (2020) and Neufeld et al. (2021) also found that stiffer hydrogels result in rounder neurons with fewer protrusions and that softer hydrogels elongate and have a higher number of dendrites. These properties seem to confer an ability to be able to more precisely modify organoids based on the structure and site being studied. A few different materials—such as alginate, agarose, chitosan, gellan gum-RGD, collagen, modified gelatin GelMa, and Matrigel—are currently in use to achieve these different physical parameters (D’Antoni et al., 2023).

#### 4.3.2 Gene editing technologies (CRISPR-Cas9 and other tools)

Gene editing technologies, such as CRISPR-Cas9, enable the ability to precisely alter the DNA of organisms. This can involve adding, removing, or modifying specific genes to explore how these genetic changes impact cell or tissue development and function. Gene editing is particularly useful for creating genetically modified organoids, offering more accurate disease models that replicate human genetic disorders at the cellular level. It is also a powerful method for studying gene function and testing potential therapies. By editing organoids, researchers can observe how genetic changes influence disease progression or tissue functionality (Driehuis and Clevers, 2017). Researchers can use CRISPR-Cas9 to knock out a gene associated with a disease to create an organoid disease model or to insert genes that are critical for developing certain features (like the expression of certain receptors or proteins). Researchers might use CRISPR to edit iPSCs to carry a mutation, and then grow those cells into disease-specific organoids (i.e., for studying AD) (Lu et al., 2021).

#### 4.3.3 Automation and high-throughput organoid generation

**Figure 1:**
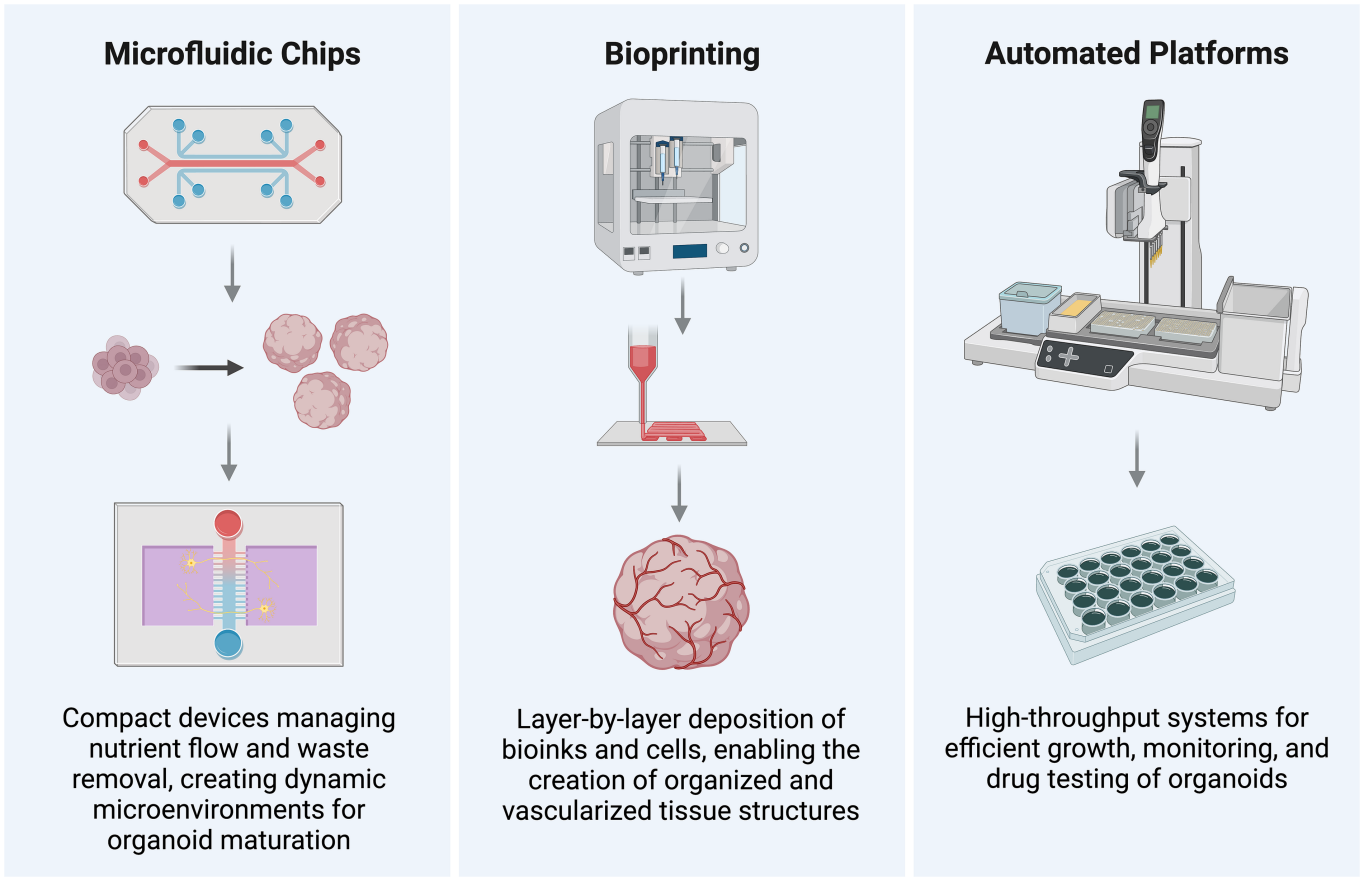
Advanced techniques enhancing brain organoid research. Demonstration of cutting-edge techniques such as single-cell omics, high-resolution imaging, and bioengineering tools (such as scaffolds and microfluidics). These methods enhance the accuracy of disease models, organoid development, and functionality. Created in BioRender. LiTe, R. (2025) https://BioRender.com/zb8h1lw.

To circumvent the issue of variability and scalability in organoid culture, specifically surrounding large-scale investigations such as drug or genetic screening for personalized medicine, researchers have employed automated platforms which combine microfluidics, 3D printing, and real-time analysis (Jiang et al., 2020; Schuster et al., 2020) (Figure 1).

Automated organoid culture platforms streamline the growth, monitoring, and analysis of organoids in a standardized way. They enable quick and efficient testing of large numbers of organoids, crucial for drug screening and studying biological processes. These platforms automate tasks like feeding and harvesting, reducing manual work, and ensuring consistent conditions increasing reproducibility. Enabling large-scale screenings of hundreds or thousands of drug candidates (in a sequential or combinational manner) in a fraction of the time, high-throughput systems help identify potential treatments for cancer, neurological disorders, and infectious diseases (Albanese et al., 2020; Renner et al., 2020; Shah et al., 2020; Louey et al., 2021).

Recently, Voitiuk et al. (2024) developed an integrated automated culture system using microfluidics for non-invasive feeding, microelectrode arrays for continuous electrophysiology monitoring, in-incubator imaging setup to maintain strict environment control, and advanced imaging for media flow feedback. This highly integrated, hands-free platform to study brain organoids presents the most advanced model that has evolved from its preceding studies where different automation techniques were multiplexed (Renner et al., 2020; Renner et al., 2021; Seiler et al., 2022).

## 5 Brain organoids for disease modeling

In recent years, brain organoids have found diverse applications across scientific fields and medicinal research due to their ability to mimic brain activity and replicate physiological tissue organization. These applications have contributed to a deeper understanding of various neurological conditions and have paved the way for potential therapeutic interventions; powerful tools that serve as models for neurological disorders, offering researchers a platform to study diseases like AD, PD, and HD in a controlled environment. By simulating the development and functionality of human brains and introducing specific genetic mutations associated with these disorders into the stem cells used to generate brain organoids, these novel technologies provide valuable insights into brain function and the underlying mechanisms of various neurological disorders, helping scientists observe how aberrant genes can contribute to disease development.

Building upon the transformative capabilities of brain organoids in elucidating neurological disorders and potential therapeutic avenues, this study aims to delve deeper into the realm of utilizing brain organoids for modeling NDs. Given the evolving landscape of research surrounding these miniature brain models, this investigation seeks to address critical research questions and objectives to further enhance our understanding of disease pathology, therapeutic strategies, and the inherent limitations of brain organoid systems.

Addressing the research question regarding the current state of research on the application of brain organoids in modeling NDs, including investigations into disease pathology, therapeutic interventions, and limitations of the model systems, the particular focus of this article is on the following diseases:

### 5.1 AD and brain organoids

**Figure 2:**
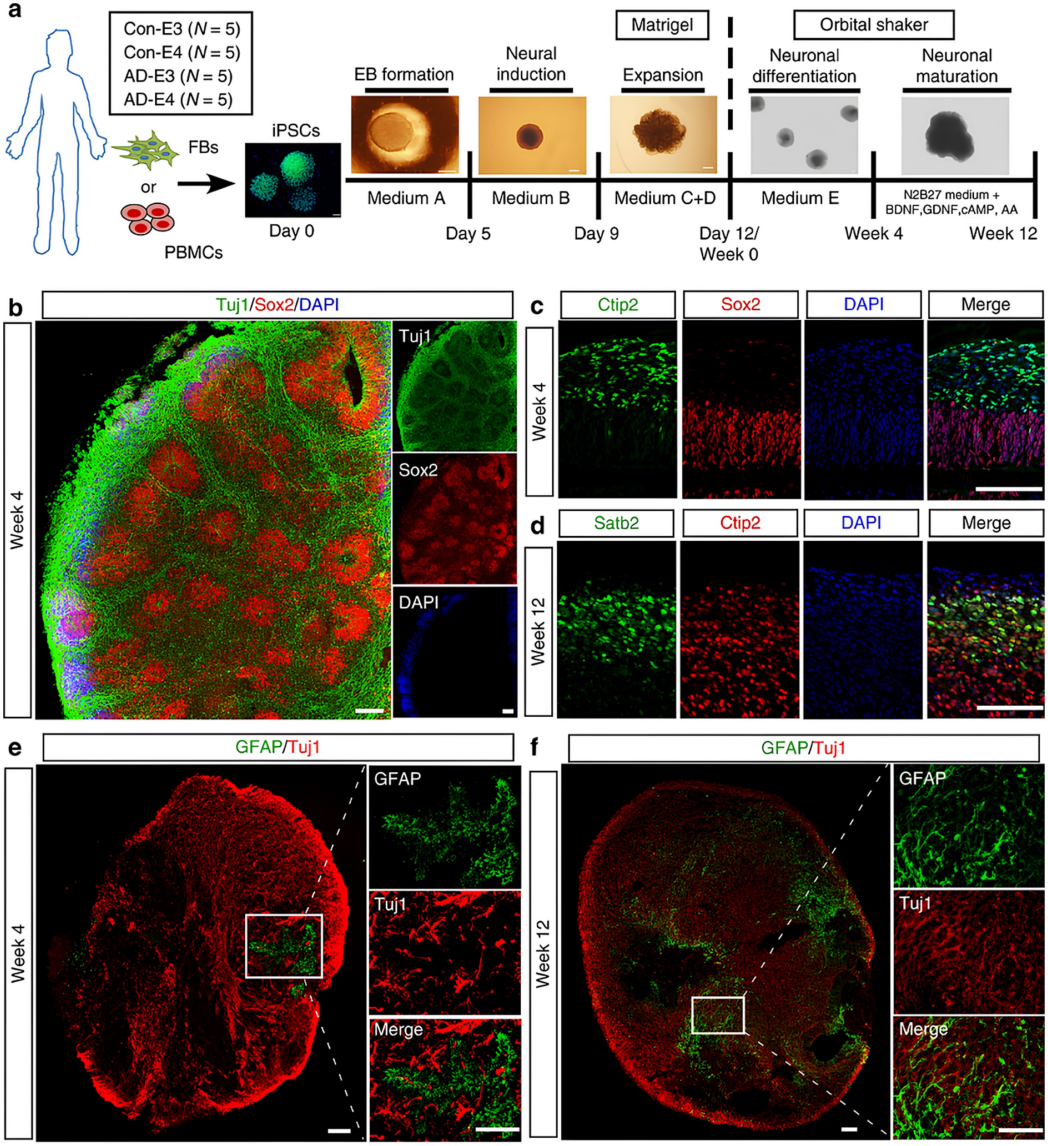
**(a)** Schematic representation of the procedures for developing cerebral organoids using hiPSCs. **(b)** Representative fluorescent images in organoids showed the ventricular zone (VZ)-like generated by new-born neurons (Tuj1, green) and neural progenitor cells (Sox2, red) after differentiation (at week 4). **(c,d)** High resolution confocal images displayed the cortical layer formation (Ctip2 stained deep cortical layer marker and Satb2 stained superficial cortical layer marker) at different developmental periods [week 4 **(c)** and week 12 **(d)**, respectively]. **(e,f)** Proliferation and migration differentiation pattern of in organoids; the differentiation pattern of astrocytes in organoids were monitored by GFAP immunostaining (astrocytic marker) at different time points [week 4 **(e)** and week 12 **(f)**, respectively]. Scale bar: 100 μm. Adapted from Zhao et al. (2020), with copyright permission under the terms of the CC-BY-NC-ND 4.0 license.

In recent years, brain organoid models have significantly advanced our understanding of ND progression, particularly in AD (Zhao et al., 2020). These models, derived from hiPSCs (Figure 2), have been instrumental in studying hallmark AD pathologies, such as the progressive accumulation of amyloidogenic amyloid-beta (Aβ) peptides and amyloid plaques, and tau hyperphosphorylation leading to neurofibrillary tangles (Dong et al., 2020; Kitahara et al., 2020; Esmail and Danter, 2021). For instance, brain organoids derived from iPSCs extracted from patients suffering from FAD exhibit elevated levels of Aβ and tau markers in comparison to healthy controls, a finding that precisely mirrors the progression of the illness. They also draw attention to how environmental variables, such as the Zika virus, can hasten the pathophysiology of AD (Conforti et al., 2018; Jorfi et al., 2018; Chang et al., 2020; Wray, 2020; Park et al., 2021; Lee et al., 2022). Moreover, APOE4 has been shown in models to have a major role in aggravating AD by decreasing Aβ clearance and interfering with cholesterol metabolism, which increases Aβ buildup and inflammation. Additionally, the APOE4 variation impairs the blood–brain barrier (BBB), which adds to the accumulation of Aβ in cerebral blood vessels (Lin et al., 2018; Ghatak et al., 2019).

**Figure 3:**
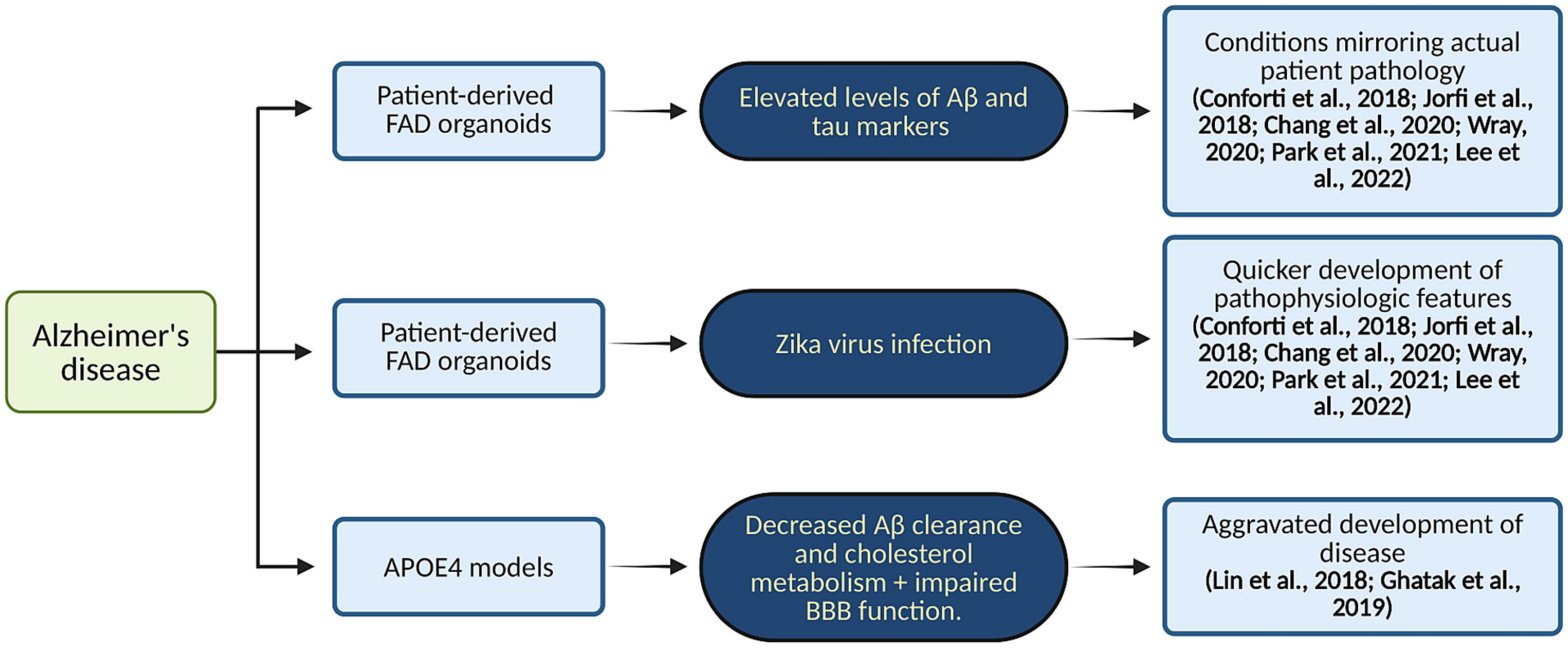
Organoid modeling of AD. This diagram illustrates patient-derived FAD and APOE4 organoids’ ability to represent actual disease pathology. Created in BioRender. Yaqinuddin, A. (2025) https://BioRender.com/vo2gq0k.

Deeper insights into the molecular mechanisms underlying the progression of AD have been gained by examining the impact of APOE genotypes on Aβ aggregation and BBB function using advanced 3D models that incorporate iPSC-derived cells (Park et al., 2018; Yoon et al., 2021). Additionally, studies showed that 3D neuro-spheroids and brain tissues from AD patients exhibit increased inflammation along with dysregulation of proteins essential for myelin production and axon development (Chen et al., 2018). Reduced inhibitory synaptic function and enhanced excitatory activity were seen in AD hiPSC-derived neurons, along with shorter neuritic processes and changed sodium currents (Chen et al., 2018; Cairns et al., 2020). Studies on oxidative stress and mitochondrial dysfunction offer important new understandings of how AD develops. These organoids’ aberrant 5-hydroxymethylcytosine (5hmC) patterns have been connected to changed neurodevelopmental gene expression, which in turn promotes the buildup of phosphorylated tau, Aβ plaques, and neurofibrillary tangles (Hernández et al., 2021; Kuehner et al., 2021). Another study emphasizes the unique significance of Aβ oligomers and their interaction with the cellular prion protein (PrPC), highlighting the vascular contributions to AD pathogenesis. Through the use of 3D neuroectodermal organoids made from iPSCs, the study showed that Aftin-5 chemical induction greatly increases Aβ accumulation (Cerneckis et al., 2023). A flow chart illustrating patient-derived FAD and APOE4 organoids’ ability to represent actual disease pathology of AD is shown in Figure 3.

### 5.2 PD and brain organoids

**Figure 4:**
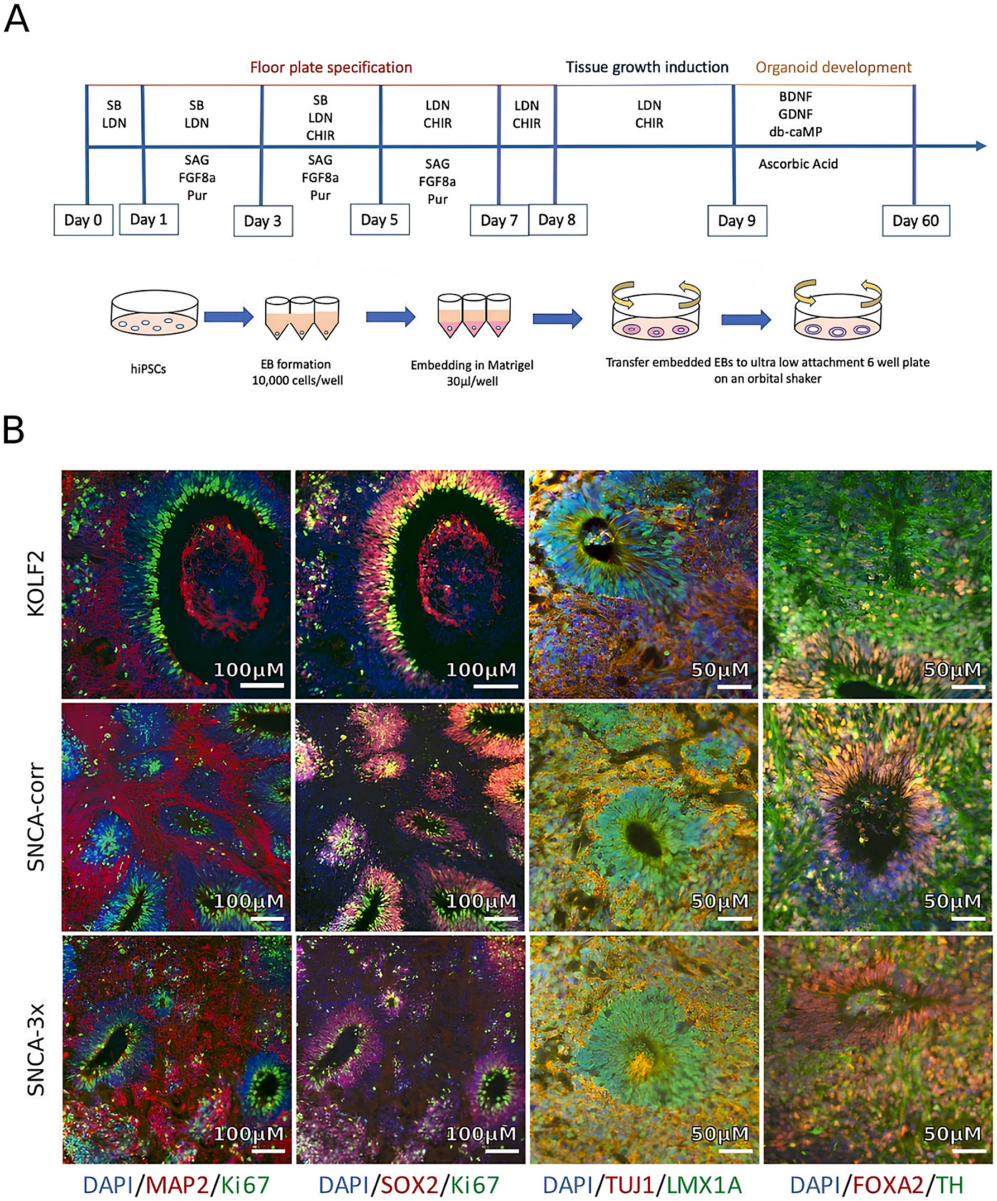
Generation and characterization of iPSC-derived midbrain organoids from Parkinson’s disease (PD) patients and healthy controls. **(A)** Schematic showing the differentiation process when utilizing induced pluripotent stem cells (iPSCs) to produce brain organoids. **(B)** Immunohistochemical evaluation following 4 weeks in culture demonstrating expression of markers specific to the midbrain: neural progenitors (SOX2), TH (dopaminergic neurons), TUJ1 and MAP2 (neuronal markers), FOXA2 and LMX1A (midbrain identity), and Ki67 (proliferation). Adapted from Patikas et al. (2023) with copyright permission under the terms of the CC-BY-NC-ND 4.0 license.

Midbrain-like organoids created from iPSCs show great potential in advancing innovative drug screening methods and therapeutic development (Figures 4A,B). These organoids can model aspects of PD pathology in the laboratory, making them valuable tools for drug discovery and personalized medicine, as well as for developing targeted treatments (Boussaad et al., 2020; Patikas et al., 2023). Research on organoids with the LRRK2 (leucine-rich repeat kinase 2) mutation—a prevalent genetic factor in both early and late-onset PD—has yielded significant insights (Kim et al., 2019; Souza et al., 2023). A study using midbrain organoids demonstrated lower levels of tyrosine hydroxylase (TH), aromatic L-amino acid decarboxylase (AADC), and dopamine transporter (DAT) while showing increased levels of caspase-3. Treatment with the LRRK2 kinase inhibitor GSK2578215A led to a reduction in phosphorylated α-synuclein accumulation, decreased dopamine neuronal cell death, and partial restoration of TH, AADC, and DAT levels (Kim et al., 2021). In addition, another study generated and studied simplified brain organoids (simBOs) from a familial PD patient with an LRRK2 mutation, noting typical PD symptoms such as elevated LRRK2 activity and reduced dopaminergic neurons. These issues were partly alleviated with the LRRK2 inhibitor PFE-360 (Chang et al., 2020; Ha et al., 2020). Furthermore, midbrain organoids with mutations in the DJ-1 gene (PARK7), associated with a highly variable form of PD, yielded significant results. The PARK7 c.192G > C mutation disrupted the binding motif for small nuclear RNA (snRNA) U1, leading to exon skipping, decreased DJ-1 protein expression, and mitochondrial dysfunction. Testing identified compounds like phenylbutyric acid and engineered U1-snRNA as effective treatments. Combining RECTAS (a kinetin analog) with phenylbutyric acid produced a synergistic effect, correcting pre-mRNA splicing, enhancing PARK7 mRNA expression, and increasing DJ-1 protein levels. This suggests that targeting splicing abnormalities to address mitochondrial dysfunction could be a promising strategy for treating sporadic PD (Boussaad et al., 2020; Piao et al., 2021). These developments offer promising prospects for therapeutic advancements in PD and open new avenues for its treatment.

Similarly, along the same timeframe, human 3D midbrain organoids (MOs) expressing the G2019S LRRK2 mutation, exhibiting abnormal localization of Ser-129 phosphorylated α-synuclein and elevated levels of light chain 3B (LC3B), an autophagy marker mainly responsible for the elimination of aggregated proteins, were derived. Therefore, the comparison of isogenic organoids differing only at the LRRK2 locus enables a more precise investigation of LRRK2-induced PD in a human model system for its early developmental stage (Kim et al., 2019; Chang et al., 2020; Ma et al., 2022). Additionally, the thioredoxin-interacting protein (TXNIP) gene was found to be upregulated in the mutant organoids, and its inhibition led to the suppression of the LRRK2 mutation, suggesting a potential contribution of LRRK2-associated sporadic PD (Kim et al., 2019; Chang et al., 2020). Furthermore, it was found that dysfunction or abnormalities in the DNAJC6 gene also result in impaired autophagy through MO (Wulansari et al., 2021). Another mutation identified in patient-derived MOs from individuals with PD was the loss of glucocerebrosidase, coupled with wild type α-synuclein overexpression, leading to the formation of Lewy body-like inclusions. In those with a genetic SNCA triplication, impaired glucocerebrosidase function also promoted the development of Lewy body-like inclusions (Jo et al., 2021). Moreover, the organoids with PINK1 deficiency showed impeded dopamine neurogenesis (Brown et al., 2021).

**Figure 5:**
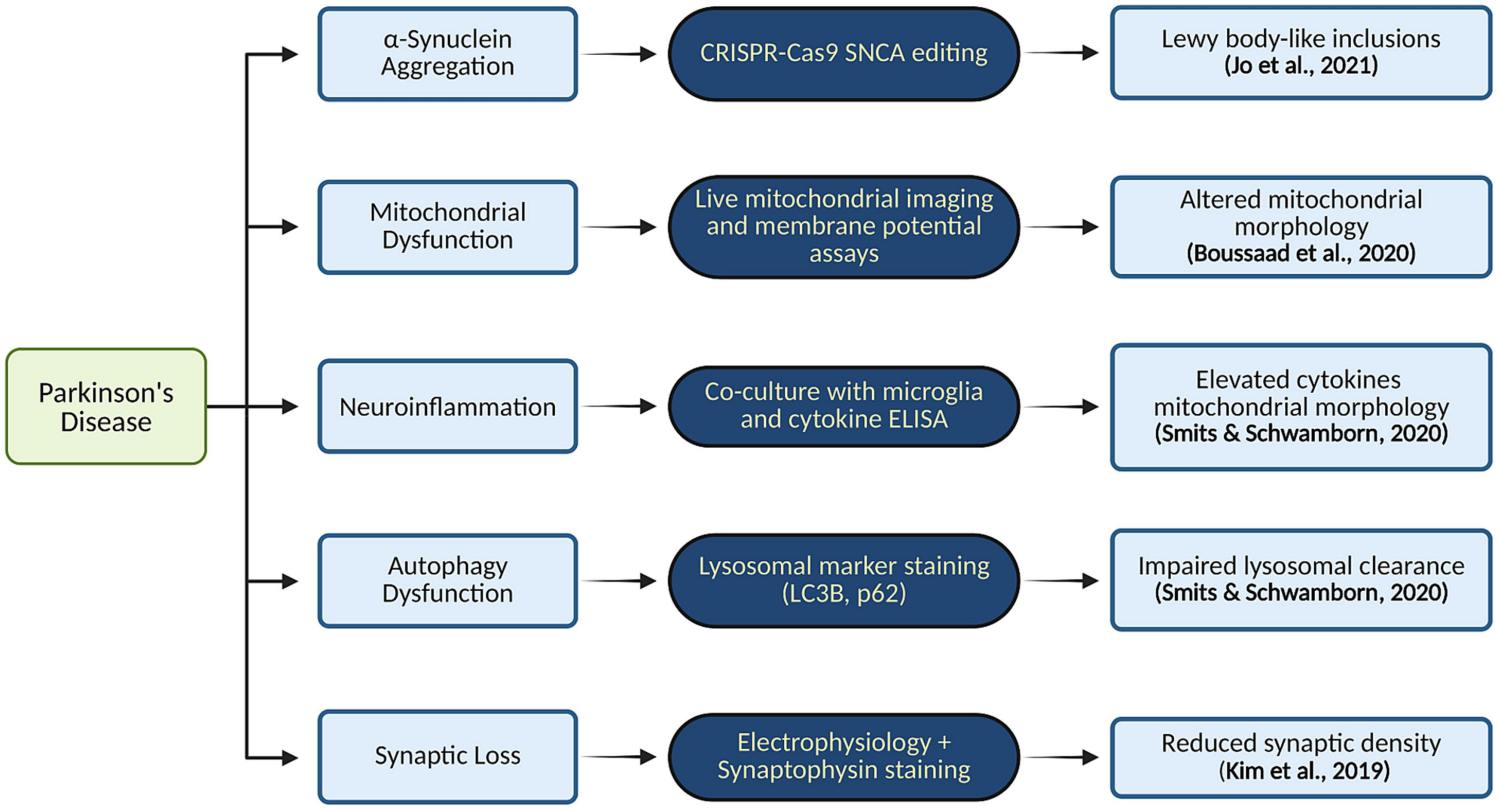
Pathway-to-phenotype mapping in PD organoid models and key molecular pathways implicated in PD. Created in BioRender. Yaqinuddin, A. (2025) https://BioRender.com/vo2gq0k.

In addition to genetic mutations, neurons within MOs have been shown to develop myelination, form synaptic connections, and exhibit normal firing patterns (Smits and Schwamborn, 2020; McComish et al., 2022). Another novel strategy involves developing DAC3.0 midbrain organoids (MOs), which closely resemble the midbrain’s structure and function *in vivo*. These organoids exhibit laminated architecture, evenly distributed mature mDA neurons, robust production of neuromelanin-like granules, and a midbrain-like cellular composition with functional glial cells and can replicate the *in vivo* pathophysiology of PD (Kwak et al., 2020). In Figure 5 we have illustrated Pathway-to-phenotype mapping in PD organoid models and key molecular pathways implicated in PD.

### 5.3 HD and brain organoids

In the case of HD, to determine the specific neurodevelopmental stages affected by the mutation and to assess whether correcting an abnormal early phenotype influences later stages of neuronal maturation, they investigated the capacity of hiPSCs to develop into dorsal cortical and ventral striatal telencephalic identities in the presence of muHTT. The findings indicate that muHTT with varying CAG repeat expansions results in inadequate down-regulation of the pluripotency marker OCT4 accompanied by reduced expression of the neuroectodermal fate determinant PAX6 (Conforti et al., 2018). The use of these iPSC-derived organoids allowed for the investigation of potential aberrant interactions between specific transcription factors and the mutant HTT protein. The study revealed a marked decrease in the expression of crucial developmental genes, such as Tbr1 and Ctip2, which are essential for the proper development and function of neurons. In another study, iPSCs generated from HD patients were differentiated into functional cortical neurons (Mehta et al., 2018). This approach allowed for the observation of various pathological features: altered transcriptomic profiles, morphological changes, and functional impairments. By comparing these differentiated neurons with those from non-diseased iPSCs, the model provided insights into the specific neurodevelopmental disruptions associated with HD (Mehta et al., 2018).

**Figure 6:**
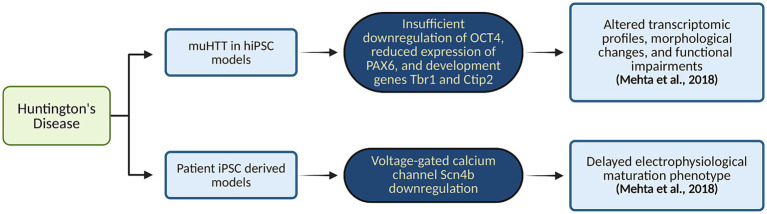
Organoid-based modeling of HD. This flowchart maps how mutant huntingtin (muHTT) expression in hiPSC-derived organoids leads to altered gene expression profiles. Created in BioRender. Yaqinuddin, A. (2025) https://BioRender.com/vo2gq0k.

Cortical cells derived from iPSCs of HD patients exhibited a notable downregulation of voltage-gated calcium channels, such as Scn4b. This reduction mirrors previous findings in HD patients and highlights a critical disruption in a mechanism essential for neurite outgrowth and axonal fasciculation. Findings also showed a delayed electrophysiological maturation phenotype, as determined by transcriptomic analysis. This delay suggests a broader dysfunction in neuronal circuitry within HD cortical neurons, reflecting compromised development and function. Moreover, the presence of muHTT in HD iPSC-derived models led to a loss-of-function phenotype within the developing cortico-striatal circuit. iPSCs from HD patients, when differentiated into cortical neurons, exhibited transcriptomic, morphological, and functional abnormalities. These alterations indicate significant deviations from normal neurodevelopmental patterns, including disrupted corticogenesis (Mehta et al., 2018). The findings from these HD-derived organoid models provide valuable insights into the molecular and neurodevelopmental disruptions associated with HD. Figure 6 maps how mutant huntingtin (muHTT) expression in hiPSC-derived organoids leads to altered gene expression profiles. By mimicking the disease’s effects in a controlled *in vitro* environment, this approach facilitates a deeper understanding of the underlying mechanisms and offers a promising platform for testing potential therapeutic interventions aimed at modifying the disease’s progression. In Table 1, we have summarized the application of brain organoid technology for brain disease modeling.

**Table 1:** Summary of brain organoid applications in modeling neurodegenerative diseases.

Number	AD phenotype/pathological finding	Modeling method	Cell/model type(s)	Publication year	References
1	Progressive accumulation of amyloidogenic Aβ peptides, Amyloid plaques and neurofibrillary tangles	Utilized a combination of molecular biology, cell culture, and histological techniques including immunofluorescence and western blot to generate and analyze COs	Human iPSCs derived from patients with familial Alzheimer’s disease, Down syndrome and healthy controls	2018	Gonzalez et al. (2018)
2	β-amyloid aggregation, tau hyperphosphorylation, neuroinflammation, microglial recruitment and axonal cleavage	3D microfluidic platform to create a tri-culture system	Genetically modified human neural progenitor cells differentiated into neurons and astrocytes, along with human adult microglia	2018	Park et al. (2018)
3	Aβ aggregates and p-tau deposits	3D tissue clearing and HCS imaging	Human iPSCs	2021	Park et al. (2021)
4	Higher Abeta aggregation and pTau seen in AD organoids compared to WT Decreased MAP2 levels in infected organoids Accelerated levels of AB in ZIKV-infected AD brain organoids Higher BACE levels in AD models compared to WT p-Tau and p-GSK3a/B (Y216/Y279) increased in AD organoids compared to WT Levels of p-PERK and p-eIF2α were higher in AD organoids compared to WT organoids PERKi treatment downregulated both Aβ and p-Tau expression in ZIKV-infected AD organoids level of Aβ production in ZIKV-infected WT organoids was increased compared to mock	Generation of embryoid bodies used for neural induction in matrigel and grown in an orbital shaker, stained with SOX2 and TUJ1	Wild-type (WT) and AD patient-derived iPSCs	2022	Lee et al. (2022)
5	Formation of plaques in VACV models slower than ZIKV models VACA AD organoids grew over time, not downregulated, unlike ZIKV models Level of p-Tau in AD organoids exposed to VACV showed no increase	Generation of embryoid bodies used for neural induction in matrigel and grown in an orbital shaker, stained with SOX2 and TUJ1	Wild-type (WT) and AD patient-derived iPSCs	2022	Lee et al. (2022)
6	Extracellular Aβ plaques and intracellular neurofibrillary tangles (NFTs) in familial AD (FAD) and sporadic AD (SAD) High Aβ42/40 ratios are found in certain 3D culture systems and cerebral organoids (COs) overexpressing FAD variants	Generation of COs using techniques like hydrogel-based scaffolds and silk fibroin-based scaffolds Other methods include overexpression of APP, PSEN1, and PSEN2 variants, as well as chemical induction of Aβ42 using Aftin-5	Human induced pluripotent stem cells (hiPSCs) differentiated into neural progenitor cells or neurons	2020	Venkataraman et al. (2020)
7	Increased levels of Aβ markers such as D54D2 and 4G8 Other AD-related pathological abnormalities such as neuroinflammation, extracellular matrix remodeling, and synaptic dysfunction	The organoids are embedded in a Matrigel matrix and cultured to develop brain region identities, including neuroectoderm and neural rosettes	Pluripotent stem cells (PSCs), which include embryonic stem cells and induced pluripotent stem cells (iPSCs) carrying familial AD (FAD) mutations	2020	Chang et al. (2020)
8	Increased levels of Aβ isoforms (Aβ42, Aβ40, and Aβ38) and accumulation of total and phosphorylated tau	Microfabricated arrays of microwells were used to generate uniformly sized neurospheroids.The neurospheroids were incorporated with a Matrigel matrix to closely mimic the 3D microenvironment of the human brain	ReN cells (genetically-engineered human neural stem cells) and human iPSC-derived neural progenitor cells (hiPSC)	2018	Jorfi et al. (2018)
9	Significantly increased Aβ42/Aβ40 ratio. The AD organoids displayed disrupted calcium homeostasis, with asynchronous calcium transients and enhanced neuronal hyperactivity compared to control organoids	3D cerebral organoids were generated with wild-type PSEN2 created using CRISPR/Cas9 technology	Human pluripotent stem cells (hPSCs) with a familial AD mutation (PSEN2N141I)	2020	Yin and VanDongen (2020)
10	The models have been used to examine the inflammatory responses at the BBB, which are relevant to AD pathogenesis, such as the effects of TNF-α and other pro-inflammatory cytokines on BBB integrity and T cell migration	Models with tubular polymeric scaffold with BMECs, smooth muscle cells, and astrocytes to mimic a 3D vessel structure and study Aβ effects	A 3D vasculogenic model composed of iPSC-derived BMECs, pericytes, and astrocytes to study the effects of different ApoE genotypes on Aβ aggregation	2021	Yoon et al. (2021)
11	APOE4 astrocytes and microglia exhibit impaired clearance of Ab42, altered cholesterol metabolism, and increased inflammatory responses compared to APOE3, leading to a higher risk of Alzheimer’s pathology APOE4 astrocytes show reduced APOE levels, hindering cholesterol transport and Ab42 clearance, while APOE4 microglia display inflammatory gene activation and compromised Ab clearance	CRISPR/Cas9 gene editing used to generate APOE4 iPSCs from parental APOE3 cells from unaffected subject	iPSCs	2018	Lin et al. (2018)
12	APOE4 variant promotes amyloid-beta (Aβ) deposition along cerebral vasculature APOE4 impairs pericytes, leading to a compromised blood–brain barrier (BBB) and hence accumulation of amyloid-beta Inhibition of calcineurin-NFAT pathway reduced APOE4-associated cerebrovascular dysfunction and amyloid-beta deposition	*In vitro* blood–brain barrier (BBB) model reconstructed using hiPSC-derived cells (pericytes, endothelial cells, astrocytes)	iPSCs	2020	Ghatak et al. (2019)
13	MBs treated with AFTIN-5 altered APP metabolism and hence an increase in AB42 levels	MB protocol reproduced with minor modifications from human iPSCs	iPSCs	2018	Pavoni et al. (2018)
14	fAD organoids exhibit premature neurogenesis due to altered Notch signaling, leading to fewer newborn neurons, which may contribute to cognitive decline in AD fAD organoids show increased production and aggregation of amyloid-beta peptides, particularly Aβ42 fAD organoids demonstrate disrupted γ-secretase activity, affecting Notch cleavage and contributing to both amyloid pathology and impaired neurogenesis	Human iPSCs with PSEN1 mutations were differentiated into cortical neurons in a 2D culture system and 3D Cerebral Organoids derived from human iPSCs with PSEN1 mutations were used to model neurogenesis and Postmortem Tissue Analysis	iPSCs	2021	Arber et al. (2021)
15	AB42: increased cell death, higher caspase activity, decreased MAP2 activity and excitatory neurons. Decrease in tau protein also observed MMP inhibitor SB-3CT increased cell death, cytotoxicity, and caspase activity in Aβ42-treated neurons Heparin, HepIII, Chabc, and HA reduced Aβ42 binding and promoted the survival of neurons in both cortical and hippocampal populations	Telencephalic spheroid model + Hippocampal spheroid model	hiPSCs	2018	Bejoy et al. (2018)
16	21 commonly dysregulated proteins in at least 2 patients Enhanced inflammation observed in AD patient brain tissues Dysregulation of proteins involved in axon development and myelin sheath formation in both 3D neuro-spheroids and AD brain tissues	iPSC derived neuro-spheroids	iPSCs from peripheral blood mononuclear cells	2018	Chen et al. (2018)
17	Increased spontaneous action potentials in AD hiPSC-derived neurons compared to controls, linked to increased excitatory and decreased inhibitory synaptic activity AD neurons exhibited shorter neuritic processes, decreased branching, increased sodium current density and faster decay of sodium currents	hiPSC-derived 2D neuronal cultures and 3D cerebral organoids carrying presenilin-1 (PS1) or amyloid precursor protein (APP)	hiPSCs	2019	Ghatak et al. (2019)
18	Large, multicellular, dense amyloid-beta (Aβ) fibrillar plaque-like formations (PLFs) upon HSV-1 infection Significant neuronal loss, reactive gliosis and increased inflammation observed upon infection Diminished neural network functionality	hiNSCs seeding into scaffold designed to mimic gray and white matter of the brain, then infused with type I collagen gel to support neurite growth	human-induced neural stem cells (hiNSCs)	2020	Cairns et al. (2020)
19	Increased production of Aβ and phosphorylated Tau	Differentiation into organoids was performed with a differ- entiation kit from Stemcell Technologies (Catalog number 08570-1) based on protocols described by (look at extra references)	iPSCs	2022	Hernández et al. (2021)
20	Aberrant patterns of 5hmC in AD organoids 5hmC modifications led to changes in neurodevelopmental genes Accumulation of amyloid-beta plaques, phosphorylated Tau, and neurofibrillary tangles	Forebrain organoids cultured from a healthy iPSC line using miniature SpinU bioreactors, using 5hmC sequencing to generate genome-wide 5hmC profiles	iPSCs	2021	Kuehner et al. (2021)
21	Ratio of amyloid-β42/40 (Aβ42/40), more significant than total amyloid-β (Aβ) levels, in inducing tau pathology	(hNPCs) overexpressing human amyloid-β precursor protein (APP) and PSEN1 with Alzheimer’s disease mutations were differentiated	Immortalized human neural progenitor cells (hNPCs) derived from human ReNcell VM cells fetuses	2020	Kwak et al. (2020)
22	Formation of Aβ oligomers in iPSC-derived neurons carrying the A246E mutation in the PSEN1 gene	iPSC-derived neurons were cultured in a 3D environment which allowed natural aggregation of Aβ	iPSC neurons	2020	Hernández-Sapiéns et al. (2020)
23	Accumulation of amyloid precursor protein (APP), increased levels of Aβ40 and Aβ42 peptides, increased Aβ42/Aβ40 ratio, tau pathology, protein aggregates, and neuronal cell death	PITRM1-knockout using CRISPR/Cas9 gene editing in human iPSCs, differentiated into cortical neurons and cerebral organoids	Human iPSC neurons	2020	Pérez et al. (2020)
24	Increased apoptosis, elevated levels of Aβ oligomers	Loss-of-function mutation in BACE2 using CRISPR/Cas9 in hPSC-derived brain organoids	Human iPSCs	2022	Luo et al. (2022)
25	Presence of AD-associated amyloid isoforms (Aβ37, Aβ38, Aβ39, Aβ40, Aβ42)	Immunofluorescence and Western Blot (WB) assays in 3D neural culture derived from iPSCs	Neurons derived from PSEN1 A246E mutant iPSCs	2020	Hasan and Trushina (2022)
26	Presence of Aβ oligomers and PrPC interaction	3D neuroectodermal organoids derived from iPSCs, with Aftin-5 chemical induction	Neurons and Neural Stem Cells (NSCs)	2018	Cerneckis et al. (2023)

This table highlights key studies utilizing brain organoids to model Alzheimer’s disease. It outlines the disease context, organoid type used, specific pathological features reproduced, and the major findings or implications for disease modeling.

## 6 Current limitations

Although progress has been made, challenges remain on the path to fully functional brain organoids. Aging represents a significant barrier to using hPSC-derived organoids to imitate AD, as it is one of the most important risk factors for developing AD, especially SAD. As highlighted by Bi et al., 2021, since cerebral organoids are genetically coded to be similar to fetal brains, they might not be “old” enough to accurately simulate the actual pathology observed in AD brains (Faravelli et al., 2020). Aging involves a number of genetic changes associated with the general change of the transcriptional profile of cells (Gerakis and Hetz, 2019; Papaspyropoulos et al., 2020). Another study highlights that cerebral organoids do not have the potential to generate mature neural networks, which are capable of modeling NDs. In the beginning, the organoids exhibit irregular activity, which gradually leads to a synchronous oscillatory pattern. Spatiotemporal variations complicate the network activity further as inhibitory neurons develop (Venkataraman et al., 2020). Due to biophysical constraints, brain organoids can only reach a maximal growth of about 4 mm (Matsui et al., 2018). Moreover, it has been demonstrated that despite the fact that exogenous endothelial cells can be incorporated into the brain organoids, the developing endothelial network may function inadequately (Pham et al., 2018). Faravelli et al. (2020) have also pointed out that brain organoids are deprived of structures that can provide orientation when they start differentiating.

Regardless of the guided or unguided approach, the resultant brain organoids present variations in their overall structure and size, total number of cells, extracellular matrix composition, presence of cavitation, and degree of permeability. The long-term culture duration spanning months involves the use of expensive media reagents and compounds the risk of culture contamination.

Without adequate vascularization, neuronal cell maturation is hindered due to insufficient oxygen and nutrient supply, leading to disruptions in synapse formation, also contributing to internal hypoxia and cellular stress that leads to necrosis and impaired cell subtype specification (Bhaduri et al., 2020; Papaspyropoulos et al., 2020). However, researchers have achieved vascularization by transplanting brain organoids into the rodent brain, facilitating the growth and invasion of host blood vessels into the human organoid. This breakthrough has resulted in enhanced cell survival due to effective blood perfusion, providing a glimpse into the future of organoid research (Cakir et al., 2019; Souza et al., 2023). Bi et al. (2021), in their study, also showed that the hemodynamics of the brain are closely related to Aβ production and tau phosphorylation.

The cerebral organoids produced by means of iPSCs from AD or Down Syndrome patients structurally resemble the human brain; however, they contain only neurons and glial cells—the oligodendrocytes are missing. Furthermore, active synapses are never formed in such organoids (Gonzalez et al., 2018). Despite representing many features of the human brain, even the best attempts yield underdeveloped structures and cells due to the lack of comprehensive studies on the molecular and physical evolution of the brain (Souza et al., 2023). The current AD cerebral organoids contain neurons and neuronal progenitors, which are ectoderm-derived but lack microglial cells—that play a role in the brain’s immunity in AD pathogenesis (Bi et al., 2021)—derived from the mesoderm. However, researchers propose that the creation of AD cerebral organoids with microglia can be achieved through the integration and co-culturing of various cell types, coupled with adjustments in culture formulations, thus offering potential solutions; by employing a co-culture strategy and incorporating iPSC-derived or primary human microglia into brain organoids, investigators seek to illuminate the intricate neuro-immune interactions that can either safeguard against or exacerbate neuronal pathologies (Nzou et al., 2018; Ormel et al., 2018; Song et al., 2019; Mayhew and Singhania, 2023).

While 3D organoids themselves seem to have placed a band-aid on the many limitations faced in previous models, namely, by providing better interactions between simulated brain regions, a limitation then arises that these organoids simulate only brain regions. Important organ-brain axis interactions have been shown to contribute to the development of AD. The gut-brain axis, brain-intestine kidney axis, and relations to heart health are a few examples (Kewcharoen et al., 2019; Lovell et al., 2019; Bi et al., 2021). Heart failure is significantly related to cognitive impairment, as suggested by Lovell et al. (2019) and Kewcharoen et al. (2019). Another restraint on the full feasibility of brain organoids is that they also lack any interactions with the sensory and motor systems, which are crucial for circuit maturation (Faravelli et al., 2020).

Furthermore, organoid-to-organoid and batch-to-batch discrepancies present significant challenges that can impede the reproducibility of brain organoid studies, thereby hindering high-throughput applications like drug screening. In addition, a critical obstacle in conducting extensive drug screening with cerebral organoids involves establishing dependable approaches for long-term preservation and cultivation, comparable to those employed for cell lines, while also tackling the protracted and time-intensive maturation process of organoids (Ma et al., 2022). In Table 2, we have highlighted disease-specific benefits and limitations of brain organoids in Alzheimer’s Disease, Parkinson’s Disease, and Huntington’s Disease.

**Table 2:** Comparative table: disease-specific benefits and limitations of brain organoids in Alzheimer’s disease, Parkinson’s disease, and Huntington’s disease.

Comparison	Alzheimer’s disease	Parkinson’s disease	Huntington’s disease
Benefits	Model hallmark pathologies: Aβ plaque formation and tau hyperphosphorylation (Dong et al., 2020; Kitahara et al., 2020; Esmail and Danter, 2021)	Enable modeling of LRRK2, DJ-1, SNCA, and PINK1 related pathologies (Kim et al., 2019; Boussaad et al., 2020; Jo et al., 2021; Brown et al., 2021)	Enables study of muHTT effects on neurodevelopment, especially corticogenesis and cortico-striatal circuit formation (Conforti et al., 2018; Mehta et al., 2018)
	Enables the study of APOE4 effects on Aβ clearance, inflammation and BBB function (Lin et al., 2018; Ghatak et al., 2019)	Allows the study of dopaminergic neuron loss, α-synuclein accumulation and lewy-body formation (Kim et al., 2021; Chang et al., 2020; Jo et al., 2021)	Demonstrates changes in OCT4, PAX6, Tbr1, Ctip2, Scn4b expression (Conforti et al., 2018; Mehta et al., 2018)
	Capture neuroinflammatory changes, excitatory and inhibitory imbalance, oxidative stress and mitochondrial dysfunction (Chen et al., 2018; Cairns et al., 2020)	Permits drug screening and evaluation of inhibitors (e.g.: LRRK2 inhibitors like GSK2578215A and PFE-360) (Chang et al., 2020; Ha et al., 2020; Kim et al., 2021)	Reveals electrophysiological delays and loss-of-function phenotypes in HD neurons (Mehta et al., 2018)
	Allows analysis of neurodevelopmental gene expression (e.g.: 5hmC) (Hernández et al., 2021; Kuehner et al., 2021)	Helps reproduce mitochondrial dysfunction and autophagy impairments (Piao et al., 2021; Wulansari et al., 2021)	Model transcriptomic, morphological and functional abnormalities using patient derived iPSCs (Mehta et al., 2018)
	Facilitates understanding of Aβ-PrPC interaction and chemical induction effects (e.g.: aftin-5) (Cerneckis et al., 2023)	Supports development of midbrain-like organoids with mature architecture and neuromelanin (Kwak et al., 2020; Smits and Schwamborn, 2020; McComish et al., 2022)	
Limitations	Lack aging related changes necessary to imitate AD, especially SAD (Faravelli et al., 2020; Bi et al., 2021)	Variability in disease expression due to mutation specific effects (Boussaad et al., 2020; Piao et al., 2021; Jo et al., 2021; Brown et al., 2021)	Focused on early development—Limited capacity to model chronic or long term disease progression (Conforti et al., 2018; Mehta et al., 2018).
	Restricted maturation and absence of fully developed neural networks (Venkataraman et al., 2020)	Limited modeling of long term disease progression (Ma et al., 2022)	
	Size constraints (max ~ 4 mm) (Matsui et al., 2018)	Technical complexity replicating a full midbrain environment (Kwak et al., 2020)	
	Absence of oligodendrocytes, active synapses and mesoderm derived microglial cells (Bi et al., 2021)		
	No modeling of organ—brain axis interactions (e.g.: gut-brain axis) (Kewcharoen et al., 2019; Lovell et al., 2019; Bi et al., 2021) (Faravelli et al., 2020)		
	Long term preservation and cultivation methods are underdeveloped making high throughput drug screening difficult due to the time-intensive process (Ma et al., 2022)		

## 7 Ethical implications of brain organoids

The advent of brain organoids raises significant ethical concerns that warrant careful examination. One pressing question pertains to the potential cognitive processes and consciousness that these organoids may possess despite their miniature size (Chen et al., 2019; Koplin and Savulescu, 2019; Hyun et al., 2020; De Jongh et al., 2022). However, it is important to note that brain organoids, despite sharing some tissue composition similarities with full-sized brains, lack the necessary organization to give rise to consciousness (Chen et al., 2019; Servick, 2020; De Jongh et al., 2022). To illustrate this, we can consider a circuit kit analogy. A fully intact and properly assembled circuit kit functions flawlessly, illuminating a bulb. Conversely, if the circuit components are randomly reassembled, the individual parts may still be discernible, but the circuit will fail to activate the bulb. Similarly, while brain organoids offer insights into different brain tissue types, they cannot generate thoughts or consciousness.

Furthermore, even if brain organoids were to exhibit a more mature brain-like organization, their limited size inherently constrains their cognitive capacities (Koplin and Savulescu, 2019; De Jongh et al., 2022). A full-sized human brain consists of approximately 86 billion neurons, whereas mini-brains contain a mere fraction of that number, around 100,000 neurons (Lancaster, 2015). The intricate interconnections and elaborate networks formed by billions of neurons in the human brain are essential for enabling higher-order cognitive processes such as perception, memory, and decision-making. Regrettably, brain organoids lack the neural complexity required to support such cognitive capabilities due to their significantly reduced neuron count.

Fortunately, concerns regarding the potential for brain organoids to evolve into larger, sentient entities are unfounded. Scientists do not anticipate the growth of larger brain organoids in the foreseeable future, primarily due to the absence of blood vessels necessary for their sustenance (Lancaster, 2015; De Jongh et al., 2022). Current brain organoid cultivation typically occurs within controlled laboratory environments, inhibiting further growth and development. Without a vascular system to supply oxygen and nutrients, the expansion of brain organoids beyond their current limited size is unfeasible.

When electrical firing patterns of the brain organoids are observed, until recently the pattern was termed inconsistent and haphazard compared to adult electroencephalogram tracings. However, Trujillo et al. (2019) compared the organoids’ firing pattern development and trajectory with that of premature infants’ brains and noticed a striking similarity underlining the usefulness of these models in studying spatiotemporal organization and formation of the neural network during early human development. Although these findings may seem as getting a step closer to mimicking a human brain, the model is still very primitive given the current limitations mentioned. Also, it is imperative to remember that at a miniature scale the neuronal firing can only give us an idea of how human neural signaling works but cannot scale to the sheer complexity of the human brain in a dish, at least for now.

Another critical factor contributing to the incapability of brain organoids to exhibit higher cognitive functions lies in their inability to interact with the external world (Chen et al., 2019; Koplin and Savulescu, 2019). Interaction with the environment plays a pivotal role in human learning, as conscious thoughts and actions emerge through intricate neural networks developed via sensory feedback. From birth, our brains constantly process sensory input, enabling us to perceive, interpret, and respond to our surroundings. In contrast, brain organoids lack sensory organs, rendering them incapable of engaging in the interactive processes that shape human cognition. Consequently, brain organoids cannot form functional networks capable of exhibiting higher cognitive functions.

On a different note, when it comes to the aspect of incorporating brain organoids into the prospect of transplantations in mouse models, there have risen other ethical concerns linked with them; one in particular being the ‘humanization’ of these species (Hyun, 2016; Chen et al., 2019). According to Chen et al. (2019), it is the ethical and moral implications that arise from the generation and utilization of neural tissues that bear a growing resemblance to the human brain, which is commonly associated with the complex cognitive abilities that define our humanity. However, this proposal in the debate on brain organoid transplantation is argued by the fact that consciousness and the ‘human-like’ traits such as self-awareness, advanced cognitive capacities, and complex emotions aren’t unique to humans alone, and are shared alike by many species, whether blood runs through its system (biological species) or not (Artificial Intelligence) (Chen et al., 2019).

## 8 Future directions

Introduced to the ground as the latest player, research and application rates of brain organoids have skyrocketed over the last decade, holding promise of its vast utilization in the coming years. However, to score that goal, organoids have to have instilled the value of being a team player; to be integrated with other currently growing technologies (Smirnova and Hartung, 2024).

Starting off with personalized medicine. Having patients with diverse genetic makeups, organoids developed from their iPSCs can be integrated into disease modeling, offering the possibility for individualized therapies (Smirnova and Hartung, 2024). This is so as to their ability to retain the fundamental traits of the developing brain and genetic makeup from individuals, thus garnering immense potential to lead the path toward personalized medicine for brain disorders (Koo et al., 2019).

Personalized organoids can also be vital for exploring molecular mechanisms and discovering novel diagnostic and prognostic biomarkers (Kelava and Lancaster, 2016; Koo et al., 2019). However, with personalization, drug testing also becomes a concern. With the need to test several drugs on each patient-specific disease, thousands of organoids would have to be produced. The arduousness of this task would mean that automation of the process would be an advantageous forte. Thus, enter SpinΩ, a scalable set of 3D printed mini bioreactors, a recent groundbreaking advancement enabling the simultaneous production of numerous organoids under diverse conditions, marking a significant step forward (Kelava and Lancaster, 2016). On that note, with the ability to print 3D organoids, researchers gain spatial control over the geometry and cell distribution, allowing for the mimicking of anatomically inspired structures within organoids (Koo et al., 2019; Smirnova and Hartung, 2024). To better assist the comprehension of individual heterogeneities, brain organoid biobanks could be instated; a gallery of different genetic and pathological snapshots of various patients, to aid in understanding brain disorders and supporting necessary therapeutic development as discussed above (Koo et al., 2019).

Multidimensional data generation via high-throughput imaging adds another area where technological integration can play a role. By leveraging Artificial Intelligence (AI) methods such as deep learning and machine learning, data processing can be accelerated, enabling the extraction of vital insights, classification of cell types, identification of morphological features, and detection of functional patterns (Koo et al., 2019; Smirnova and Hartung, 2024). With that, gates to a hybrid technology open; Organoid Intelligence (OI)—models that can exhibit cognition and learning (Smirnova and Hartung, 2024). Having many physiological processes difficult to perceive and thus, understand, OI systems provide a unique window into complex physiological processes, enabling direct experimental exploration of neuronal signaling and network dynamics (Smirnova and Hartung, 2024). As technological advancements progress rapidly, OI models have the potential to simulate human brain complexity by improving cell connections and myelination, promising precise representations of cognition and advanced functions alongside early proof-of-concept studies showing responsiveness to stimuli and learning abilities driven by neurophysiological adaptations (Koo et al., 2019; Smirnova and Hartung, 2024).

Concurrently, Brain-Computer Interfaces (BCIs) are also starting to rise, allowing the communication between external devices and the brain with wide applications. While primarily targeting the restoration of sensory-motor functions in cases of paralysis through brain implants, they also hold promise for enhancing memory, accessing knowledge, and enabling seamless control of technologies through thoughts. With the intertwining of organoids into this field, safer BCIs could be developed, guided by the organoids’ ability to fabricate neural structure and function (Smirnova and Hartung, 2024). Symbiotically, BCI sensors could analyze organoid activity patterns during both development and disease modeling, revolutionizing research, education, and the synergy between soul and code (Smirnova and Hartung, 2024).

## 9 Conclusion

Brain organoid models have proven to be invaluable tools in understanding the pathological mechanisms of major NDs like AD, PD, and HD. Their ability to replicate *in vivo*-like conditions enhances their potential in drug discovery and personalized medicine. However, significant limitations remain, such as the immaturity of organoids, incomplete vascularization, and the absence of significant cell types like microglia and a lack of sensory input, which impede their ability to replicate complex neural networks and age-related pathologies. Ethical concerns regarding cognitive capabilities and regarding transplanting human-like tissues into animal models deserve careful attention, especially as such procedures raise questions about the potential ‘humanization’ of other species and prompt the need for updated ethical frameworks. Despite these challenges, advancements in technology, including patient-specific organoids, automation in production, and the establishment of biobanks, hold promise for individualized therapies. Looking ahead, the integration of brain organoids with emerging technologies like SpinΩ bioreactors and 3D printing promises scalable, reproducible production, essential for high-throughput drug testing and biobank development. Combined with artificial intelligence and high-content imaging, organoids can enable more refined, multidimensional data analysis, unlocking new insights into disease mechanisms. The integration of high-throughput imaging, AI, and BCI can further enhance the utility of organoids, potentially leading to OI that simulates aspects of learning and cognition. This convergence could not only revolutionize biomedical research but also help advance neurotechnology and human-machine interactions. To fully harness the potential of brain organoids, future research should adopt a holistic approach that not only focuses on improving their complexity and addressing ethical considerations but also advances cell-type diversity, vascularization, and more dynamic brain-environment interactions through strong interdisciplinary collaboration to overcome existing barriers.

## Glossary

AD: Alzheimer’s disease
PD: Parkinson’s disease
HD: Huntington’s disease
hPSC: Human pluripotent stem cell
ESC: Embryonic stem cells
iPSC: Induced pluripotent stem cell
3D: Three dimensional
MeSH: Medical subject headings
ND: Neurodegenerative disease
Aβ: Amyloid-beta
SAD: Sporadic Alzheimer’s disease
FAD: Familial Alzheimer’s disease
APP: Amyloid precursor protein
PSEN1, PSEN2: Presenilin
BBB: Blood brain barrier
APOE4: Apolipoprotein E gene
LRRK2: Leucine-rich repeat kinase two
PINK1: PTEN-induced kinase 1
2D: Two dimensional
CAG: Cytosine-adenine-guanine
HTT: Huntingtin
muHTT: Mutant HTT
EB: Embryoid bodies
ASC: Adult stem cell
PSC: Pluripotent stem cell
Tumoroids: Tumor-derived organoids
VEGF: Endothelial growth factor
FGF2: Fibroblast growth factor 2
5hmC: 5-hydroxymethylcytosine
PrPC: Cellular prion protein
TH: Tyrosine hydroxylase
AADC: Aromatic L-amino acid decarboxylase
DAT: Dopamine transporter
MO: Midbrain organoid
LC3B: Light chain 3B
TXNIP: Thioredoxin-interacting protein
AI: Artificial intelligence
OI: Organoid intelligence
BCI: Brain-computer interface
